# Work Stress, Mental Health and Validation of Professional Stress Scale (PSS) in an Italian-Speaking Teachers Sample

**DOI:** 10.3390/healthcare9111434

**Published:** 2021-10-25

**Authors:** Pierpaolo Limone, Roberto Zefferino, Giusi Antonia Toto, Gianfranco Tomei

**Affiliations:** 1 Department of Humastic Studies, University of Foggia, Arpi Street, 71122 Foggia, Italy; pierpaolo.limone@unifg.it; 2 Department of Medical and Surgical Sciences, University of Foggia, Luigi Pinto Street, 71122 Foggia, Italy; roberto.zefferino@unifg.it; 3 Department of Psychiatric and Psychological Science, University of Rome “Sapienza”, Queen Elena Street, 00161 Rome, Italy; gianfranco.tomei@uniroma1.it

**Keywords:** work stress, mental health, Italian professional stress scale

## Abstract

This study aimed validate the Italian version of the Professional Stress Scale (PSS). A questionnaire was translated into Italian and administered to two sample groups. The first group (*n* = 200) was the control group and the second (*n* = 1137) the experimental group. The participants in the study were students enrolled in a special needs training teacher course or a specialization course that aims to train support teachers. The study conducted two analyses; factor and reliability analyses. The factor analysis utilized the Kaiser-Meyer-Olkin (KMO) test which had a result of 0.925 for the scale; this was above the acceptable value of 0.7. The research studied 33 items and the BTS was significant for the 33 items scale (χ2 (528) = 4353.508, *p* < 0.001). Moreover, five eigenvalues greater than 1 were identified in the data, whereas the total variance explained was 63.7%. The reliability test utilized the Cronbach’s Alpha score (0.936) of the scale and the value is calculated based on the response of 1106 individuals. The value is well above the value of 0.80, which indicates a high internal consistency level of the different items of the scale. This study showed that the Italian version of the PSS is a reliable and valid measure that can be used for research and clinical purposes.

## 1. Introduction

Stress is often associated with negative experiences or notions of distress and negative effects that are related with the incapacity to deal with them. Every job can produce a certain level of stress which can affect individuals at all levels in the organization, from employees to managers to senior leaders. If stress is occasional it does not appear to be harmful, but problems often arise when it is chronic. Some of the sources of stress at work include interactions among employees, the workload, personal responsibility, and conflicts between home and work. Stress is known to cause adverse effects on health, physically and mentally [1,2]. For instance, it can lead to an escalated anxiety, substance addiction, burnout and depression. Employees who feel stressed at work are highly likely to get involved in unhealthy behaviors like poor diet, smoking, drug abuse, and alcoholism. Reports show that excess work stress leads to approximately 120,000 deaths and a total of approximately $190 billion in health care costs annually [3] around the world. This is about 5–8% of the healthcare spending of national economies, which is a result of high direct expenditures (about $48 billion), lack of insurance cover (about $40 billion), and conflict ($24 billion) [4]. Work stress can worsen an existing mental health problem, thus making it harder to control. Common mental and physical health issues and stress can exist independently, meaning people can be stressed about work and at the same time experience physical changes like high blood pressure with no depression or other mental health conditions. They may also have depression without experiencing stress. The effects of work stress include reduced job satisfaction, absenteeism, poor delivery of services and higher mortality rates.

Employees today are experiencing greater challenges than ever because of company strategies, policies, restructuring, technological advancements, and deadlines that lead to high levels of professional stress. Professional stress occurs due to a discrepancy between the demands at the workplace and the capacity of an individual to meet these demands [5,6]. Professional stress greatly affects productivity, mental, physical, behavioral, and emotional health among professionals. The Professional Stress Scale (PSS) is a self-reporting technique for establishing sources of stress for professionals. It is utilized to determine the degree of stress experienced by professionals in the healthcare workplace (specifically referencing mental health professionals who work in schools such as school psychologists) and in the school environment [7,8]. This paper aims to discuss work stress, mental health and validate the Professional Stress Scale. For this purpose, the 33-item PSS scale, which includes four subscales, was administered to 200 individuals in a cool group and 1137 individuals in an experimental group. It had a Cronbach’s Alpha score of 0.936 and KMO score of 0.925.

Most jobs may promote work related stress, particularly nowadays it involves the healthcare sector [9,10,11]. There are few studies carried out on mental health professionals, whose work is often considered less interesting, however, lots of research shows that these professionals may display high vulnerability to stress. Reports show that psychiatrists seem to have the highest rates of suicide among healthcare practitioners [12,13]. Other authors report that mental health professionals experience a wide range of problems which lead them to drop out of training [14]. According to [15], there is a perceived distress level of 59% of clinical psychology trainees, which is higher than in other groups. If the stresses of mental health professionals have to be mitigated, it is important to systematically analyze the stressors that cause them. Some studies show that mental health professionals face different stressors from other professionals in other healthcare sectors. Mental health studies show that besides being susceptible to stressors that are inbuilt in the workplace, mental health workers tend to face more challenges. For instance, a descriptive literature review reveals that the two primary sources of stress in healthcare facilities are patient contact, and organizational and administrative factors. According to the research conducted by Travers and Firth-Cozens [16], some of the causes of stress in the workplace include lack of resources and shortage of personnel, along with the major one being violent experiences with patients. While most of the problems in psychiatry field seem to be common to all health workers, the impacts may be aggravated by the intrinsic nature of the job. As such, Ref. [17,18] show that there are unique problems in this group that should be addressed. Thus, there is a great need for more research on the specific stressors faced by mental health workers. Hellman et al. [19] established five key stressors related to the therapeutic role of mental health workers in the public and private sectors. These factors include scheduling, professional uncertainty, personal degradation, work overload, and sustaining a therapeutic relationship. In a study of psychologists, Cushway and Tyler [20] noted that factors like self-doubt and client distress were stress factors. Additionally, other factors like organizational issues and workload were revealed. The coronavirus has changed the way people live and work. The study by Irawanto et al. [21] revealed that working from home, work-life balance, and work-related stress have significant effects, both direct and indirect, on job satisfaction. In fact, COVID-19 has changed working conditions due to social distancing policies. As many workers have begun to use new technologies at work, the study by Oksanen et al. [22] probed the potential effects of social media communication (SMC) stress on work. The results indicate a disparity in the resilience of workers during remote work and highlight the need for organisational support [23]. The study by Hong et al. [24], on the other hand, examined the associations between work overload, parental stress, work-family conflict, and job satisfaction during COVID-19. Seven hundred and eighteen kindergarten teachers participated in the study.

Labour resources can buffer the deleterious effect of adverse work environments. Stress reactivity can be an important work resource on a personal biological level. Deng et al. [25] examined how stress responsiveness interacts with work environments in predicting work burnout. Social stress at work appears to accelerate the loss of resources over consecutive working days. Elfering et al. [26] analyse workplace social stressors and resources possessed. The study by Wang et al. [27] aimed to investigate the prevalence of burnout as well as anxiety, depression and stress in resident physicians and evaluate the effects of an online psychological intervention on the mental health status of physicians with a high degree of burnout. The work-related well-being of an employee is to some extent related to the work environment perceived by colleagues rather than absolute [28,29]. It found that perceiving colleagues as having higher or lower demands than themselves is associated with lower job satisfaction and higher levels of emotional exhaustion. Therefore, the processes of social confrontation regarding job requests can influence the well-being of employees.

There are contexts such as education, services and the helping professions where nothing material is built and such immateriality of the work product can unsettle the individual. The lack of a material aspect makes, for some, the work objectionable because the results are not visible and immediate. In these areas, if the worker has not made strategic alliances, his or her work life can be very problematic. For this reason, in order to better evaluate the dynamics related to work-stress, the need to validate the PSS tool in Italian emerged. It also it seemed useful and interesting to validate the tool with educational professionals: individuals who often have difficulty in immediately recognizing the fruits of their labor.

In order to verify the presence of a work-related stress situation for these professionals, the Professional Stress Scale (PSS) which investigates through appropriate questions certain parameters such as workload, difficulty in working with others, lack of resources, conflicts with colleagues and superiors, self-esteem and home/work conflicts was administered. Although the standardized version has a good diffusion, in the specific Italian context the need arises for the validation of tools that make research in work-related stress a sector of greater depth [30,31,32]. Today’s work with the use of technologies, multitasking, e-mails, the Internet insert today’s worker into a “network” in which at times they risk being harnessed.

The present study thus aimed to validate the Italian version of the Mental Health Professionals Stress Scale, developed in 1996 by Cushway and colleagues. The Mental Health Professionals Stress Scale (MHPSS) measures stress for mental health professionals using the self-report method and identifying sources of stress. The expected relationships between the scale and between the criterion measures—the General Health Questionnaire, a symptom check list, job satisfaction, self-reported stress level and quality of social support—were demonstrated. The results also provide evidence for the discriminant validity of the subscales to measure different aspects of the stress experience [20].

The Italian version of MHPSS was used, prior to the present validation, in two studies by Zefferino and colleagues in 2006 [33] and 2009 [34].

The aim of the 2006 study was to identify the presence of sources of stress in an urban police emergency team and the causes of such stress using the PSS test and biomarkers such as salivary cortisol and interleukin 1β. In the 2009 study Zefferino investigated stress using a double approach: (i) a psycho-diagnostic test able to show psychological effects and (ii) a kit test able to measure salivary markers of stress as cortisol and interleukin 1β.

The present study responds to a pressing need to structure a protocol for the diagnosis and treatment of psychophysical stress within the services of the University Hospital of Foggia.

## 2. Methods

### 2.1. Study Design

Often, the subject does not know at the end of the day what his work consisted of. There are claims that in areas such as education, services and the aid professions nothing material is built, and the immateriality of the product of work is something that can upset the individual, as the lack of a material aspect makes work critical. The Mental Health Professionals Stress Scale (MHPSS) is a self-assessment method for identifying sources of stress for mental health professionals. The 42-item scale, which includes seven subscales, was administered to 154 clinical psychologists and 111 mental health nurses. MHPSS was found to have good internal consistency (α = 0.87 for clinical psychologists; α = 0.94 for mental health nurses). Preliminary evidence suggests that the simultaneous validity of MHPSS is good. The modified mental health Professional Stress Scale (PSS) is used to assess self-perceived work-related stress [31]. This research included a validation of the Italian Version of Professional Stress Scale [32,33]. The questionnaire consists of 33 questions and detects the frequency of occurrence of certain stressful events using the following 4-value scale: 0 = never happened, 1 = doesn’t happen usually, 2 = happens occasionally, and 3 = happens to me.

Emotions, thoughts, workload, relationships with colleagues and superiors, and the ability to reconcile professional and personal life are investigated. The total score is equal to the sum of the scores obtained for each item. Higher values indicate a greater degree of work-related stress.The items and the answer alternatives are easy to understand. In two previous experiments, the questionnaire was applied to a specific category of workers (an urban police team) who, by answering a specific question, showed maximum understanding of the questions. Furthermore, the questions are of a general nature and, therefore, are free from content specific to any subpopulation. To examine the proposed PSS, Zefferino et al. [34] conducted an explorative factor analysis (EFA) and a confirmatory factor analysis (CFA). The authors reported that there was support for validity of the Professional Stress Scale.

These results showed that the factors present an acceptable validity and reliability. In addition, the instrument was shown to have adequate convergent validity with theoretically related constructs. All constructs exhibited composite trait reliability levels that exceeded 0.7 [34], ranging between 0.87 and 0.95.

### 2.2. Sample

Subjects participating in our study were teachers enrolled in a TFA support specialization course, a course that aims to train support teachers. The sample was selected in order to investigate the level of perceived stress in teachers and validate the scale on this sample. The authors and research team evaluated the intentional sample through the technique of heterogeneous sampling. This type of sampling is intended to provide a wide range of cases relevant to a particular phenomenon or event. The purpose of this type of sample design is to provide as much information as possible about the event or phenomenon under consideration. In the case of the present research, it was deemed useful to analyze the sample of teachers enrolled in the TFA support specialization course.

The study involved samples from two groups, a control group (*n* = 200), and an experimental group (*n* = 1137), but the results are based on the information obtained from the responses of 1106 respondents.

An attempt was made to make the experimental group as large as possible to reduce the influence of non-obvious differences between the subjects, and the aim was also to simultaneously reduce the probability of incurring a first and second type of error.

To build the Italian version of a foreign instrument, it is necessary to start with data collection to verify the validity and reliability of the tool for the Italian context. Once these have been verified, the standardization sample can be collected. The standardization sample should normally be proportional to the population at which the test is aimed and present samples similar in characteristics to those present in the original manual.

The control group was made up of 200 people who represent in percentages the same demographic and socio-cultural characteristics (educational qualification, geographical origin) of the experimental group (25% belonging to each grade of kindergarten, primary, secondary, and secondary school second degree and maintaining the same gender ratio).

The pilot study is developed in the Italian context, where the initial training course for teachers is online and distributed nationally; therefore, teacher interviews from all areas of Italy (67% from southern Italy) were included. By pilot study, we mean it is an exploratory test survey to demonstrate that the Italian version of the PSS test can work so that it can subsequently be used on a large scale. It is therefore presented as a feasibility study intended to test the questionnaire to guide the application of the test on large dimensions.

The Italian version of the questionnaire was administered to teachers on the initial training course to support teachers at the University of Foggia (*n* = 1106). The data were broken down by demographic profile, response processing and educational level. The data was provided via a Google form in December 2020 during the COVID-19 state of emergency. Using the online form made it possible to receive results in real time and to quickly view a summary.

### 2.3. Data Collection Instrument

The research utilized questionnaires to collect information and the professional stress scale to determine the factors contributing to stress in the workplace.

### 2.4. Ethics

The research study complied with the general ethical principles of the Declaration of Helsinki and was approved by the research team’s University Institutional Review Board, protocol code 40979-III.11 and approved on 6 August 2021 issued by La Sapienza University of Foggia.

### 2.5. Statistical Analysis

The research performed a t-test to assess the differences between the means. A chi square test was also conducted to establish the statistical significance of the subscales. To evaluate the external validity, the research made comparisons using the PSS, to determine the level which occurrences are considered stressful. The PSS version that was used was a 33-item tool. The research utilized the Cronbach’s Alpha score, which was 0.936, to determine consistency. The study also conducted a factor analysis using the KMO test which was 0.925.

To establish if the collected information was suitable for the analysis, we applied the Kaiser-MeyerOlkin (KMO) measure of sampling adequacy and BTS tests. The KMO value was acceptable (0.943) and the BTS was significant (χ2 (528) = 18,361.702, *p* < 0.001). As such, the data was relevant for factor analysis; thus, a principal components analysis was performed. The results showed that the majority of the items showed the factor loading values of 0.5 and above, whereas items 6, 29, 31, and 32 have factor loading values of 4 or above, which are acceptable values in research. Since the chi-square value is less than 3 (2.360), it shows that the model was adequate.

## 3. Results

### 3.1. Sample Description

Data was taken from Google modules in reference to a sample of 1137 people. The experimental group consisted of school teachers enrolled in the TFA support course as students. The subjects belong to the 5th cycle of the University of Foggia, of which 85.7% are women and 14.3% men. Although there are only slight numerical differences with respect to the degree of origin, the most representative degree was lower secondary school (29% of the participants). The age varied between 20 and 60 years.

A total of 1106 respondents completed the questionnaire. The 33 items were evaluated through the PCA extraction method in which the majority of the items had factor loading values of 0.5 and above, while a few had 4. The KMO test result value was 0.943, an acceptable value of above 0.7. The BTS was significant for the 33 items scale (χ^2^ (528) = 18,361.702, *p* < 0.001). Also, 6 eigenvalues greater than 1 were identified in the data, while the total variance explained was 60.8%. Internal consistency Cronbach’s a for the entire sample was found to be 0.936.

### 3.2. Reliability Analysis

Table 1 shows the Cronbach’s Alpha score (0.936) of the scale and the value is calculated based on the response of 1106 individuals. The value is well above the baseline value of 0.80, which indicates a high internal consistency level of the different items of the scale. Moreover, Table 2 shows the item statistics that include the values of the mean and standard deviation of all the items of the scale.

### 3.3. Factor Analysis

The 33 items of the scale were examined through the PCA extraction method. As shown in Table 3, the majority of the items showed factor loading values of 0.5 and above, whereas items 6, 29, 31, and 32 have factor loading values of 4 or above, which are accepted values in research [35,36]. The demographic items were not included in the factor analysis.

#### Extraction Method: Principal Component Analysis

**Figure 1:**
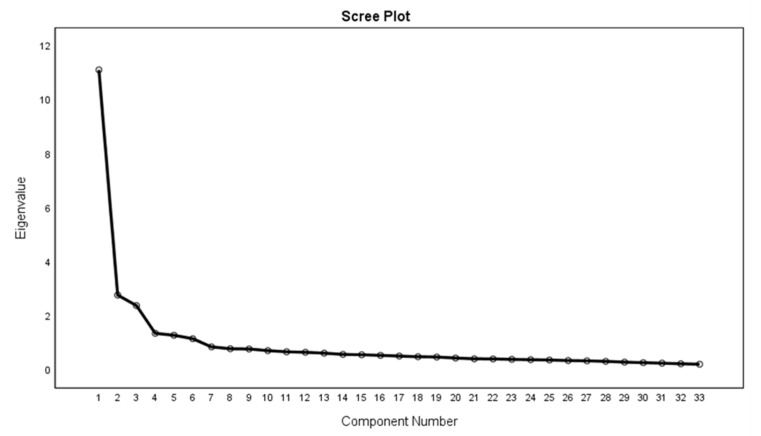
Screen Plot.

The KMO test result value was 0.943 for the scale, which is also well above the acceptable value of 0.7. The BTS was also significant for the 33 items scale (χ^2^ (528) = 18,361.702, *p* < 0.001) (Table 3). Moreover, six eigenvalues greater than 1 were identified in the data, whereas the total variance explained was 60.8%. Besides, a scree plot (Figure 1) also showed the six identified eigenvalues of 11.1, 2.8, 2.4, 1.4, 1.3, and 1.2.

## 4. Discussion

To verify the presence of a situation of work-related stress, the PSS was administered, which investigates through appropriate questions some parameters such as workload, difficulty in working with others, lack of resources, conflicts with colleagues and superiors, self-esteem and home/work conflicts. The effectiveness of these scales for measuring stress has been studied in various mental health settings, ranging from nurses, to university students, to public administration workers [37,38]. Edwards and Burnard [39] revealed an excessive level of workplace stress for mental health nurses. The most frequently reported sources of stress were administrative and organizational concerns, patient issues, heavy workload, interprofessional conflict, financial and resource issues, professional self-doubt, home/work conflict, staffing levels, changes in the health service, maintaining standards, lecturing and teaching, long waiting lists, and poor supervision. 

Lee et al. [40] identified, by analyzing a sample characterized by psychologists, nurses, and social workers that for depression and anxiety, that scores appeared slightly different across professional groups. In fact, nurses and social workers showed significantly higher total scores than clinical psychologists, and there were significant differences in subscale scores among professionals. Results and reviews conducted to date have suggested that the scale was a useful measure and predictor of stress [41]. Specifically, in this study, the PSS was administered to professionals in the educational setting; indeed, it was found that there are contexts such as education, service, and helping professions in which the immateriality of the work product can upset the individual. For this reason, in order to better assess the dynamics related to work stress, the need emerges to validate the PSS tool in Italian.

Challenges, beyond the dimensions already highlighted, could also lead to depression, high blood pressure, and fatal coronary conditions [42]. As such, it is vital to conduct the occupational stress scale because it determines the magnitude of stress experienced by professionals in the healthcare workplace. The results of this study show that the PSS has good characteristics and can be used in future research.

## 5. Limitations of the Study

The data provided in this study do not represent the whole Italian employed population, as the study focused on mostly teachers rather than on workers in all sectors of the economy. The Italian healthcare setting comprises of many mental health professionals; thus, a more representative sample should be examined for future studies. Although the sample is not broadly representative of different work contexts, we believe it can be used in other work contexts as well since the sample was large (*n* = 1.106). The non-random sampling technique used to recruit the sample could have contributed to selection bias and lack of representativeness.

Thus, future research should consider sampling workers from all sectors to attain more extensive results. Lastly, the study does not categorize and discuss individual subscales; thus, the results are linked to only two classes of workers (police in the experimental phase and teachers in this validation), and they should also be extended to other categories to generalize the clinical results in the Italian context. Therefore, future observations should categorize the subscales accordingly for easy analysis and interpretation of the results.

## 6. Conclusions

The results of this research indicate that the Italian version of the Professional Stress Scale has excellent features, and thus, it qualifies to be used for future research. The initial evidence concerning the usage of the PSS is quite intriguing and inspiring, even though there is still a need for more information and analysis. Thus, reliability analysis and more validation of the PSS are worthwhile objectives for future studies. Besides providing evidence of the validity of PSS with other professionals, a study like this could improve the understanding of the causes of stress for professionals in the workplace.

## Figures and Tables

**Table 1:** Cronbach’s Alpha.

Cronbach’s Alpha	Cronbach’s Alpha Based on Standardized Items	No of Items
0.936	0.936	33

**Table 2:** Item Statistics.

Item	N	Mean	Std. Deviation	Scale Mean If Item Deleted	Scale Variance If Item Deleted	Corrected Item Total Correlation	Cronbach’s Alpha if Item Deleted	Loadings
1	1106	2.30	0.794	45.44	287.383	0.403	0.935	0.578
2	1106	2.19	0.873	45.56	284.850	0.450	0.935	0.615
3	1106	1.90	0.901	45.85	283.228	0.489	0.935	0.538
4	1106	1.89	0.940	45.86	283.835	0.447	0.935	0.680
5	1106	1.75	0.942	45.99	281.950	0.507	0.935	0.551
6	1106	1.38	0.868	46.36	282.821	0.524	0.934	0.433
7	1106	1.54	0.916	46.20	278.040	0.655	0.933	0.595
8	1106	1.29	0.981	46.45	278.168	0.603	0.934	0.626
9	1106	1.42	0.900	46.32	277.754	0.677	0.933	0.699
10	1106	1.37	0.907	46.37	277.327	0.686	0.933	0.741
11	1106	1.42	0.935	46.32	276.562	0.689	0.933	0.716
12	1106	1.36	0.918	46.39	276.822	0.694	0.933	0.737
13	1106	1.35	0.948	46.39	277.218	0.658	0.933	0.664
14	1106	1.62	0.990	46.12	279.618	0.552	0.934	0.601
15	1106	1.79	0.956	45.96	279.093	0.590	0.934	0.734
16	1106	1.32	0.980	46.43	280.245	0.538	0.934	0.680
17	1106	1.43	0.939	46.31	278.564	0.620	0.933	0.660
18	1106	0.97	0.883	46.77	280.074	0.610	0.934	0.604
19	1106	1.01	0.949	46.74	279.644	0.577	0.934	0.596
20	1106	0.46	0.755	47.29	287.191	0.434	0.935	0.583
21	1106	1.19	0.926	46.55	280.800	0.555	0.934	0.531
22	1106	1.42	0.905	46.33	281.707	0.538	0.934	0.519
23	1106	0.99	0.833					0.558
24	1106	1.25	0.927					0.756

**Table 3:** Eigen Values, KMO & BTS Tests.

Test							
Eigen Value	11.1	2.8	2.4		1.4	1.3	1.2
Percentage of Variance	33.7	8.4	7.2		4.1	3.9	3.5
Total Variance Explained				60.8			
Kaiser-Meyer-Olkin Measure of Sampling Adequacy				0.943			
Approx. Chi-Square (BTS)				18,361.702			
Df				528			
Sig.				0.000			
